# Annotated receipts capture household food purchases from a broad range of sources

**DOI:** 10.1186/1479-5868-6-37

**Published:** 2009-07-01

**Authors:** Simone A French, Melanie Wall, Nathan R Mitchell, Scott T Shimotsu, Ericka Welsh

**Affiliations:** 1 University of Minnesota, Division of Epidemiology & Community Health 1300 South 2nd St, Suite 300, Minneapolis, MN 55454, USA; 2 University of Minnesota, Biostatistics, 420 Delaware St SE, Minneapolis, MN 55455, USA

## Abstract

**Background:**

Accurate measurement of household food purchase behavior (HFPB) is important for understanding its association with household characteristics, individual dietary intake and neighborhood food retail outlets. However, little research has been done to develop measures of HFPB. The main objective of this paper is to describe the development of a measure of HFPB using annotated food purchase receipts.

**Methods:**

Households collected and annotated food purchase receipts for a four-week period as part of the baseline assessment of a household nutrition intervention. Receipts were collected from all food sources, including grocery stores and restaurants. Households (n = 90) were recruited from the community as part of an obesity prevention intervention conducted in 2007–2008 in Minneapolis, Minnesota, USA. Household primary shoppers were trained to follow a standardized receipt collection and annotation protocol. Annotated receipts were mailed weekly to research staff. Staff coded the receipt data and entered it into a database. Total food dollars, proportion of food dollars, and ounces of food purchased were examined for different food sources and food categories. Descriptive statistics and correlations are presented.

**Results:**

A total of 2,483 receipts were returned by 90 households. Home sources comprised 45% of receipts and eating-out sources 55%. Eating-out entrees were proportionally the largest single food category based on counts (16.6%) and dollars ($106 per month). Two-week expenditures were highly correlated (r = 0.83) with four-week expenditures.

**Conclusion:**

Receipt data provided important quantitative information about HFPB from a wide range of sources and food categories. Two weeks may be adequate to reliably characterize HFPB using annotated receipts.

## Background

A major trend during the past two decades has been a shift among households from purchasing foods from grocery stores and eating home-prepared meals to purchasing prepared foods from full-service and fast food restaurants, coffee shops and other stores [1-6]. In 2000, almost half of the US household food dollar was spent at eating-out food sources [3,6]. It is estimated that by 2010, 53% of the US household food dollar will be spent at eating-out food sources [3-6]. According to national individual dietary intake surveys, about 57% of US adults eat away from home on any given day [3-6]. Away from home food refers to food that is obtained from outside the home, such as restaurants, fast food places, vending machines and cafeterias [4-6]. Food obtained from away from home sources comprises about 25% or more of daily energy intake [4,5]. To date, however, research that examines household food purchases has focused only on grocery store food and beverage purchases [1].

The term "household food purchase behavior" (HFPB) reflects a broader conceptualization of the household food environment [see ref [1] for a review], and refers to all foods and beverages purchased by the household from all sources, including grocery stores, restaurants, convenience stores, coffee shops and department stores [1]. HFPB is important to measure because it may be an important influence on individual energy intake and dietary quality, and possibly excess weight gain and obesity [1-6]. It is especially important to include this broader range of food sources, given their significant contribution to household food expenditures and individual energy intake. The HFPB is an intermediate level variable between the neighborhood retail food environment and individual dietary intake. It may exert direct effects on individual intake through food exposure and availability [1], and indirect effects through its role as a mediator of the neighborhood retail food environment [7-12].

Research progress in this area is hindered due to the lack of measures of the HFPB that capture the detail and range of food and beverages purchased and their sources [1]. The household food environment has been measured in previous research using home food inventories, food purchase records, grocery store receipts and bar code scanners [1,12]. These methods have focused on grocery store food and beverage purchases and have not captured eating-out food sources in any detail. For example, the most comprehensive food purchase record studies are from household consumer expenditure studies, or household budget surveys [13,14]. These national surveys are collected at regular intervals by several European countries to estimate price indexes, but they also provide detailed data on household food expenditures [1,13-20]. For example, the Data Food Networking initiative is a multi-country study that pooled and analyzed data from household budget surveys of several European Union countries [13,14]. However, detailed data about the sources, types or quantities of foods and beverages purchased from eating-out sources were not published [1-6,12-20].

Food purchase receipts are a method that has been used to describe household food purchases that have the potential to provide detailed information about food purchases from eating out sources, such as restaurants, coffee shops and convenience stores. Receipt data can provide detailed information about food sources, food items, spending and quantities, and thus when combined with individual-level or neighborhood-level data, can enhance understanding of possible links between the neighborhood retail food environment, household food purchases, and individual dietary intake.

The present paper describes the development of a measure of HFPB using annotated receipts. The receipt measure was developed to assess HFPB as part of a community-based household weight gain prevention intervention. The primary purpose of this paper is to describe the method, its development, and potential application in community-based nutrition research. To help illustrate the usefulness of the method, this paper will describe the food purchase sources (e.g. grocery, convenience store), and specific types of foods purchased (e.g. unprocessed whole foods, prepared foods). Specific study questions are: 1) What percent of the household food purchases are from grocery stores, restaurants, and other retail food sources? 2) What percent of household food purchases are for specific types of foods (e.g. fruits, vegetables, prepackaged prepared foods); 3) How many weeks are sufficient to reliably capture HFPB (item counts, quantities, expenditures)? While the answers to these questions are specific to the sample of households in the present study, the method and approach to quantifying the answers to these questions shows the value of the methodology and provides an example of how to collect, code and analyze these data. The method can then be used in further studies to examine associations with theoretically important variables to better understand influences on household food purchases and individual dietary intake.

## Methods

### Study population and household characteristics

Data for the present study were collected as part of a community-based household weight gain prevention intervention conducted in Minneapolis, Minnesota, USA in 2007–2008. The study was a group-randomized trial, with households the unit of randomization, intervention and analysis. Data presented here are cross-sectional baseline data only. Households (n = 90) were recruited from the community using a variety of methods. Fliers and brochures were distributed in local community centers, libraries, grocery stores and schools. In-person recruitment was also conducted at community events, health fairs, and after-school programs. Interested households contacted study staff and were screened for eligibility. Study eligibility criteria were related to the weight gain prevention intervention aims. Eligibility criteria included the presence of at least one adult and one child in the household, residence in a private house or apartment within 15 miles of the university, and willingness to be randomized to active intervention or control group. The requirement that households reside within 15 miles of the University ensured that household members were close enough to the University to conveniently attend the intervention sessions. The University of Minnesota IRB approved the study.

Households completed a face-to-face clinic visit during which all household members completed weight and height measures, and survey self-report measures of eating behaviors and physical activity. Following the clinic visit, a home visit was scheduled. During the home visit, the research staff person trained the primary household shopper on the receipt collection and annotation protocol (described below). The receipt collection training required about 10–15 minutes. A home food inventory was completed by the research staff during the home visit. Home visits required between 45 minutes and 2 hours, depending on the amount of food present in the household.

### Receipt protocol training and data collection

During the home visit, a trained research staff member explained the receipt collection and annotation protocol to the primary household shopper. He or she was given a binder with step-by-step instructions and example receipts to practice with during the home visit. The research staff person reviewed the practice examples with the primary shopper to clarify how to annotate the receipts. Primary shoppers were instructed to query other household members about any food purchases they made during the week. The binder included annotation sheets, a mailing schedule and stamped return envelopes. Primary shoppers were instructed to collect and annotate receipts and mail them to study staff on a weekly basis. Receipt annotation sheets queried the primary shopper for information about the food source, food item, quantities, cost and source (name and type). The specific name of the food source was written in and a box was checked to indicate the type of food source. Source types were the USDA food source categories used in the CSFII [21], and included store, restaurant, carry out, cafeteria, vending machine, mail order, bar/tavern and other source. The primary shopper was instructed to complete a receipt annotation sheet regardless of whether a receipt was available (e.g. if food was purchased from a vending machine or convenience store and no receipt was provided). For simplicity, we will use the term receipt to refer to receipt annotation sheet hereafter.

Study staff followed up with the primary shoppers after the first week of receipt collection. If receipts were late or improperly annotated, the study staff called to remind the primary shopper and to answer any questions. The most common annotation difficulty was with estimating portion sizes from restaurant and carry out food sources. Food models were not used for portion size training with the primary shopper. Primary shoppers were instructed to estimate portion sizes to the best of their ability if portion size information was not available on the food package, for example in restaurant settings. For many chain restaurants, food and beverage portion sizes could be determined by research staff members during the receipt coding and editing process by consulting the restaurant web site nutrition facts information (see receipt coding, below). About 60% of the households required at least one reminder prompt. These simple prompts consisted of a telephone voicemail or an email to participants, and required very little staff time to complete.

### Receipt coding and data entry protocol

**Figure 1:**
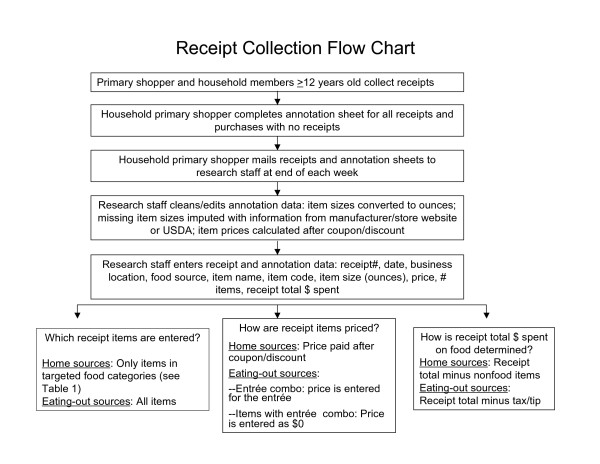
**Receipt collection flow chart**.

The receipt coding and data entry protocol is shown in Figure 1. When received, the receipts were numbered, edited and coded by a trained research staff member using a defined written protocol, then entered into a database. The receipt annotation sheets completed by the primary shopper were used as the basic unit of data collection and the actual receipts from the source location were used only for editing purposes. Start date for the receipt collection was entered and receipts were numbered. The business name and address was entered for each receipt. Total receipt expenditure was entered. The USDA food source categories were verified for accuracy by research staff based on the store name and location. Research staff further classified sources in the "store" category into a grocery store chain or market, a convenience store/gas station, or a super-center. Foods and beverages were coded into one of the defined food categories (see Table 1 and described below). Information on quantities (ounces) and price was entered for each food and beverage item that was included in the pool of items entered.

**Table 1:** Description of targeted food categories for receipt coding

	**Example Items**	
**Food Category**	**Home**	**Eating-Out**
**Fruits and Vegetables**		
Fruits	Apples (2 lbs)	Apple slices (4 oz)
Vegetables (not potatoes)		
Fried	Onion rings (frozen 1 lb)	Onion rings (3 oz)
Not fried	Broccoli (fresh 1 lb)	Green side salad
Potatoes		
Fried	French fries (frozen 1 lb)	French fries (6 oz)
Not fried	Raw white potatoes (5 lb)	Mashed potatoes (4 oz)
**Snacks and Sweets**		
Snacks	Corn chips (16 oz)	Corn chips (5 oz)
Sweets	Doughnut (1 lb)	Doughnut (2 oz)
**Entrées and Sides**		
Prepackaged Entrée		
Less than 500 calories	Frozen pasta entrée (single serving 10 oz)	NA
500 calories or more	Frozen pizza (20 oz)	NA
Entrée-eating out	NA	Cheeseburger; burrito
Side foods	NA	Refried beans; coleslaw
**Beverages**		
Non-caloric beverages	Bottled water (1 L)	Diet cola (16 oz)
Sugar-sweetened beverages	Carbonated sweetened cola (1 L)	Carbonated sweetened cola (16 oz)
Fruit/Vegetable juice	100% orange juice (1 L)	100% orange juice (16 oz)
Alcoholic beverages	NA	Beer (1 pint)
Specialty beverages	NA	Milkshake (20 oz)
		Frappuccino (20 oz)

NOTE: Home NA categories are **NOT **used for coding home items. Eating-out NA categories are **NOT **used for coding eating out items.

Food categories are listed in Table 1. In the initial piloting of the receipt annotation method, all foods were included in the coding process, including uncooked grains, prepackaged side foods and desserts requiring preparation, and baking ingredients (e.g. rice dishes, dry cake or dessert mixes, flour). However, it became clear that the categorization process for the foods would be complex and that the annotation would be prohibitively time-consuming and burdensome for participants. A decision was made to limit grocery store annotation to foods and beverages targeted by the intervention because of their potential link to excess weight gain. The specific targeted food categories were: fruits, vegetables, prepackaged snacks and sweets, prepackaged entrees, and beverages.

For eating-out food receipts, all of the foods and beverages purchased were included in the annotation and coding (not only the targeted food categories listed above for grocery store receipts). The rationale for coding all items purchased from eating-out sources was that little data are currently available about non-grocery store food and beverage purchases by households. The eating-out receipt data therefore provided a unique opportunity to describe and examine this broader universe of household food purchases in detail.

A hierarchy was implemented for the eating-out food and beverage receipt coding. If possible, eating-out food and beverages were coded into the categories used for the grocery store purchases. However, additional food categories were added to capture the broader range of foods purchased from eating-out sources. The additional food categories were used only for those eating-out items that did not fit into the initial food categories generated for the grocery store receipts. For example, lettuce salad fit into the existing category "vegetable" when purchased from a restaurant. However, egg roll was coded as "side" because none of the existing grocery store categories captured egg roll.

All entrée foods from eating out sources were coded as "entrée eating-out." This separate entrée category was necessary because there was insufficient information available to accurately classify these eating-out entrées as "less than 500 kcal" or "500 kcal or greater" as was done for prepackaged entrées purchased from grocery stores. In addition, one goal of the intervention was to promote less frequent eating-out. Thus, for the purpose of the intervention and its evaluation, all eating out entrées were similarly targeted for reduction in frequency of purchase and consumption. When entrée foods were served with side foods, these foods were separately annotated and coded. For example, a burrito with beans and rice was coded as "entrée eating-out," and two "sides." Additional beverage categories were generated to capture the purchase of alcoholic beverages and specialty beverages (e.g. smoothies, lattes, specialty coffee or yogurt beverages).

Foods purchased for non-household members were not included in the food coding or price recording. Item prices were recorded without tax and tips and after discounts when identifiable. Prices for combination meals were recorded by item if available. If only one price was charged for a combination plate, the price was attributed to the entrée and side items and beverages were listed as zero cost.

Only a subset of the home source food and beverage purchases were recorded. Thus, a true denominator for total household food and beverage expenditures cannot be generated by summing expenditures on each item in the database. Neither can the total amount from the receipt be used, as it may include non-food items (e.g. shampoo, medicine, paper products). To best estimate total household expenditure, a hybrid method was used. For store purchases, if 90% or more of the total cost was for food and beverage items (as estimated by research staff during data entry), then the pre-tax receipt total was retained. In all other cases, the receipt total was replaced with the sum cost of the food and beverage items. Note that this method only affects home food purchases. The receipt data collection method was designed to capture all of the household's eating out food purchases, so summing the cost of the food and beverage items generated a true denominator for total eating out purchases.

### Statistical analysis and summary variables computed

Food and beverage purchases for all receipt annotation sheets were included in all analyses (n = 2,483 annotation sheets). Summary variables were calculated from the receipt data. Quantity in ounces, number of items (food line items) and expenditures in dollars were summed within food and source categories. The number of receipts returned was summed by household by week. The total number of days a household returned a receipt that was from a restaurant or carry out food source was computed. To examine purchases within specific food and beverage categories, the percent of expenditures for specific food categories within the home and eating-out sources were computed using the total number of line items.

Stability in the number of food purchases and in the types of food purchases across time was examined using correlations. Associations were examined for total receipts, receipts by source and receipts by specific food categories, such as fruits and vegetables. Week-to-total correlations were computed to examine whether one-, two-, or three-week receipt collection was similar to four weeks. High week-to-four-week correlations show that a shorter time period of receipt collection may provide stable estimates of food purchase amounts and types.

Seasonality was examined to observe whether food and beverage purchases varied according to the month of the year in which households enrolled in the study. These analyses showed few differences in the expenditures on different types of foods and beverages, or in the sources of food purchases, by month of study enrollment and are not further discussed.

## Results

### Receipt completion rates

One hundred six households completed the initial baseline clinic visit. Of these, 90 households completed four weeks of receipt collection and were enrolled in the study. Thus, the drop out rate from clinic visit to receipt collection was 15% (90/106 = 85% completers).

A total of 2,483 receipt annotation sheets were returned by the 90 households. Of these, 1,892 (75.2%) included receipts plus annotation sheets, and 591 included annotation sheets only (no receipt). A total of 9,300 food line items were included in the receipt annotation sheets.

### Household descriptive characteristics

The demographic characteristics of the households were self-reported by the main respondent for the household. Ninety-three percent of the primary shoppers were female, and 78% were white. Households on average were comprised of four people (1.8 adults (range 1–4) and 2.0 children (range 1–5)). The most common configuration was two adults and two children (51%). The primary shopper was on average 40.8 years (sd = 7.3 yrs), 64% were married or living with their significant other; 25.8% had more than a college degree. Reported household income was distributed as follows: 35% ≤ $45,000 per year; 30% between $50,000 and $95,000; and 35% ≥ $100,000 per year. The average body mass index for the primary shopper was 29.7 kg/m2 (sd = 7.2 kg/m2). Household primary shoppers reported eating from a carry-out restaurant 2.3 times and from a sit down restaurant 1.1 times during the preceding week. On average, household primary shoppers reported that the household ate six meals together during the preceding week.

### Number of receipts collected, food sources and food expenditures

Table 2 shows the number of receipts collected and expenditures from 90 households during the four-week data collection period. A total of 2,483 receipts were collected, about 27.5 receipts on average per household, and about 6.9 receipts per week per household. The number of receipts was split almost evenly between home sources (45%) and eating-out sources (55%). The greatest number of receipts was from stores, followed by carry-out restaurants. Within the carry-out sources (n = 756 receipts), 30.3% were from burger chain restaurants, 11.3% were from coffee shops, 10.8% were from sandwich chain shops, 9.8% were from Mexican style restaurants, and 7.2% were from pizza chains.

**Table 2:** Number of receipts, food expenditures, and food sources among 90 households

	**Receipt Count**		**Monthly Cost/HH**		
**Sources**	**Total # of receipts**	**Total % of receipts**	**Mean ($)**	**Std. ($)**	**Max ($)**
**Eating Out**	1,372	55.3	168.65	152.88	849.68
Carry-out	756	30.5	67.61	61.11	306.68
Restaurant	345	13.9	80.61	106.07	568.75
Cafeteria	135	5.4	7.56	18.39	98.00
Other	70	2.8	9.62	23.12	143.90
Vending	51	2.1	0.86	2.23	15.00
Bar/Tav	15	0.6	2.40	7.63	42.20
**Home**	1,111	44.7	336.46	213.96	1,252.08
Store	795	32.2	286.07	209.26	1,252.08
Convenience store/Gas station	266	10.7	24.25	38.45	234.12
Super-center	48	1.9	24.82	69.62	509.19
Mail	2	0.08	1.32	10.39	96.18

**TOTAL**	2,483	100	505.11	289.05	1,630.45

Average total food and beverage expenditures (defined above) were $505 per household per four-week period, or about $126 per week per household. Average expenditures from eating-out sources totaled $168 per four-week period, or about $42 per week per household. Average expenditures from home sources totaled $336 per household per four-week period, $84 per week. Stores comprised the majority of home food expenditures, while eating-out expenditures were comprised mainly from restaurants and carry-out places. The average weekly expenditure among households from burger chains was $4.17; coffee shops $1.07; sandwich chain $1.50; Mexican style food $1.65; and pizza chain $0.65.

### Food category purchases: home foods and eating-out

Table 3 shows the distribution of 9,300 receipt food items after being coded into one of the food categories. For each category, the absolute number of foods, the proportion of total items, average household total expenditures, average household total ounces and cost per ounce are shown. For number of items purchased, entrées from eating-out sources comprised 16.6% (n = 1,546) of the total recorded food items across all receipts, proportionally the largest single category of the annotated items. Sweets (14.4%), vegetables (not fried; 13.1%) and fruits (11.0%) were the second, third and fourth most frequently purchased items.

**Table 3:** Food categories: percent of all items purchased, and average household (n = 90) expenditures and ounces

**Sum across all HHs**			**Monthly Average per HH**		
**Food Category**	**# Items**	**% Items**	**Cost ($)**	**Ounces**	**Cost/Ounce**
**Fruits and Vegetables**					
Fruit	1,020	11.0	32.07	373.50	.0858
Vegetables (not potatoes)					
Not fried	1,221	13.1	34.85	257.37	.1354
Fried	26	0.3	0.86	1.64	.5226
Potatoes					
Not fried	157	1.7	3.40	60.38	.0562
Fried	403	4.3	4.17	34.62	.1205
**Snacks and Sweets**					
Snacks	772	8.3	23.68	118.96	.1990
Sweets	1,337	14.4	43.89	237.55	.1847
**Entrées and Sides**					
Prepackaged Entrée					
< 500 kcal	212	2.3	8.26	38.64	.2137
> 500 kcal	259	2.8	13.93	86.66	.1607
Eating out entrée	1,546	16.6	106.91	204.22	.5235
Eating out side orders	339	3.6	7.24	22.11	.3275
**Beverages**					
Non-caloric beverages	483	5.2	12.73	526.27	.0242
Sugar-sweetened beverages	846	9.1	18.95	558.89	.0339
Fruit/Vegetable juice	281	3.0	9.77	184.55	.0529
Eating out alcoholic beverages	88	0.9	7.50	15.96	.4698
Eating out specialty drinks	310	3.3	8.38	53.67	.1561

NOTE. The two left columns show the distribution of the 9,300 line items on all receipts collected at baseline according to the category of food as which they were classified. For example, of the 9,300 line items, 11%, or 1,020 of these were fruit. The third and fourth columns from the left show the average per household cost and ounces for each food category. For example, the average amount of fruit purchased per household was 373.50 ounces, and the average fruit cost was $32.07. The right column shows the cost per ounce for each food category.

For dollars expended, eating out entrées represent the largest category of food expenditures ($106.91) per household per month. The main types of foods purchased within the entrées when eating-out, based on counts of items, were sandwich/wrap (22%); burgers/hot dogs (17%); Mexican foods (10%); pizzas (10%) and breakfast foods (10%) (data not shown in table). The combined fruit and vegetable (not fried) categories represent the second largest category of monthly food expenditures ($66.92 fruit and vegetables not fried; $74.49 including fried and not fried potatoes). It should be noted that expenditures for vegetables and potatoes were underestimated because the expenditure for these items was coded as zero when they were included with the eating out entrée price. Snacks and sweets combined represented the third largest food expenditure category. Households spent an average of $67.57 per household per month on snacks and sweets.

Of the beverage purchases, sugar-sweetened beverages were the largest dollar expenditure, followed by non-caloric beverages such as water or diet soft drinks. It should be noted that expenditures for sugar-sweetened beverages and non-caloric beverages were underestimated because expenditures for these items were coded zero when they were included with an eating out entrée price. Expenditures for 100% fruit or vegetable juices and for specialty drinks eating-out (e.g. smoothies, lattes, yogurt drinks) were modestly lower than the sugar-sweetened beverages and the non-caloric beverages.

By definition, 100% of the eating-out entrées, side dishes and eating-out alcohol items were purchased from eating-out sources. Fried potatoes (81%) and other fried vegetables (89%) were also primarily purchased from eating-out sources. By contrast, 99% of fruits, 78% of vegetables, 86% of fruit and vegetable juices, 89% of snacks, and 78% of sweets were purchased from home food sources (data not shown in Table).

Food costs per ounce are shown in the right-most column of Table 3. Eating-out entrée was the most expensive food category, but it includes items not individually charged, such as side foods and beverages included with the cost of the entrée. Fried vegetables and alcoholic beverages were expensive but infrequent purchases. By contrast, fruits and vegetables were less expensive than sweets and snacks. The least expensive per ounce was non-caloric and sugar-sweetened beverages.

### Stability of week-to-week purchases within food categories

Table 4 shows the correlations between mean four-week expenditures and one-, two- and three-week expenditures for total and individual food categories. As expected, the correlation with four-week totals increases as the number of weeks increase. It is of interest to examine how well shorter time periods correlate with the four-week total. For example, the correlation between average fruit expenditure in weeks one-, two- and three- with the four-week fruit expenditure total is very high at 0.95, while the correlation between fruit expenditure using only week one data and the four-week fruit expenditure total is lower, at 0.75. Overall, the two-week expenditure was highly correlated with the four-week expenditure, averaging 0.83, across all the food categories (ranging from 0.40 for fried vegetables to 0.95 for specialty drinks). Three-week correlations were even higher, on average 0.92 with four-week total expenditures. These data suggest that receipt collection for two or three weeks is highly correlated with four-week receipt data.

**Table 4:** Correlations between four-week receipt total costs, with one-, two-, and three- week receipts among 90 households

	**Total Four-Week Correlation With:**		
**Food Category**	**One Week**	**Two Weeks**	**Three Weeks**
**Fruits And Vegetables**			
Fruit	.75	.89	.95
Vegetables (not potatoes)			
Not fried	.76	.89	.96
Fried	.22	.40	.61
Potatoes			
Not fried	.56	.80	.93
Fried	.72	.84	.94
**Snacks And Sweets**			
Snacks	.60	.86	.94
Sweets	.80	.85	.94
**Entrées and Sides**			
Prepackaged Entrée			
< 500 kcal	.62	.80	.96
> 500 kcal	.23	.78	.94
Eating-out entrée	.78	.91	.96
Side orders	.86	.91	.97
**Beverages**			
Non-caloric beverages	.70	.88	.97
Sugar-sweetened beverages	.74	.88	.92
Fruit/Vegetable juice	.74	.81	.92
Alcoholic beverages	.74	.84	.92
Specialty drinks	.90	.95	.97

**TOTAL**			
Mean	.67	.83	.92
Median	.74	.85	.94

### Participant feedback about receipt collection completeness and burden

Feedback was collected from the primary household shopper who completed the receipt collection using a structured questionnaire following the final four-week receipt collection at the 12-month follow up. The survey queried the primary shopper about the extent to which the receipt records represented their grocery store, other store, restaurant and fast food purchases; the proportion of purchases captured by the annotated receipts, and the time and effort required to complete the receipt annotation and collection procedure.

Sixty-four percent of the household primary shoppers reported that almost all of their own total purchases were captured by their receipt annotation. However, only 40% reported that almost all of the other adults' purchases were captured, and 41% reported that almost all of the adolescent youth purchases were captured. An additional 24% reported that about half to more than half of their own purchases were captured; 23% reported about half to more than half of the other adults' purchases were captured, and 29% reported about half to more than half of the adolescents' purchases were captured.

Eighty-eight percent of household primary shoppers reported that the receipt records represented well the type of foods and beverages that they usually purchased. Eighty-eight percent also reported that grocery food and beverage purchases were well-represented, and 75% reported that restaurant, fast food and other store food and beverage purchases were well-represented.

Most household primary shoppers (61%) reported that the amount of time required for receipt collection and annotation was not a problem. Twenty percent reported the receipt collection was a small problem and 18% reported that it was a significant problem. A similar proportion of primary shoppers reported that low motivation was not a problem (62%), and that other household members were cooperative (65%). While only 4% cited better in-person training, and only 10% cited more follow-up from staff, 49% of household primary shoppers reported that more financial incentives or other rewards would be helpful in the receipt annotation collection protocol.

## Discussion

This paper described the development of an annotated receipt measure to collect and quantify information about household food purchases in a sample of 90 diverse households from the community. The results showed that it is feasible to collect annotated receipts for a four-week period from community households in which the primary shopper receives brief training, feedback and follow up telephone prompting from research staff.

Receipt data provided important detailed information about household food purchases from a wide range of sources, including grocery stores, restaurants and carry-out places. To date, studies describing household food purchases have only included grocery store food purchases, and have not included detailed information about eating-out food purchases [1-6,13-20]. The inclusion of eating-out sources is of great interest given that about half the household food expenditure is on foods and beverages from away from home sources [1-6]. According to data from the US National Restaurant Association, 47.9% of US household food expenditures are spent eating out [3]. Estimates from the 2000 US Consumer Expenditure Survey show that approximately half of the household food dollars are spent on eating-away sources such as full-service restaurants and fast-food chains [6].

In the present study, about 33.6% of the total household food receipt costs were for eating-out foods. Estimates of expenditures on eating-out sources from receipt data in the present study are slightly lower than national estimates [6]. Several factors could be contributing to these results, including the self-selected sample characteristics and the targeted nature of the receipt collection. For example, households that volunteered for a healthy lifestyle study initially may be more likely to eat at home more frequently compared to other households. Households with children may also be more likely to eat at home more frequently. National data show that households with no children are more likely to eat out at full-service restaurants and fast-food chains compared with households with children [6]. However, the reported of frequency of eating out at restaurants and fast food places in the present study was similar to national estimates [6].

The receipt collection methodology implemented in the present study also may have contributed to the lower eating-out expenditure observed. First, receipts from eating-out episodes in which other people paid for the meal, such as social or business meals, were not captured. Second, the household primary shopper may not have received comprehensive information from all household members about their individual eating-out expenditures. Some data supporting this hypothesis was observed in the present study. For example, the number of eating-out receipts declined over the four-week period, suggesting that frequent small purchases from coffee shops or fast food places may not have been completely captured. Also, 75% of the household primary shoppers reported that their restaurant and fast food purchases were well-represented in the receipt data, compared with 88% reported for grocery store food and beverage purchases. Thus, participant burden for four weeks of receipt collection may contribute to incomplete receipt collection. The stability data show that the data collection period could be reduced significantly, from four weeks to two weeks. This shorter time period for receipt collection could improve completeness of the receipt data collection and reduce participant burden while maintaining data quality.

Annotated receipt data provided detailed, quantitative information about the sources of the food items, the number of items, expenditures and ounces. These three methods for quantifying food and beverage purchases provide slightly different insights into the HFPB. Expenditures on food and beverages reveal what households are choosing to pay for, and quantities data reveal the amount of food acquired and available to the household. Comparison of expenditures and quantities can provide insights into household priorities and constraints with regard to food expenditures and food quality. For example, in the present study, clearly the least expensive food per ounce was sugar-sweetened and non-caloric beverages. Also, contrary to popular belief, fruit and vegetables were on average less expensive per ounce than prepackaged sweets and snacks [22-26]. These data do not support the idea that healthful foods cost more than less healthful foods, at least at the aggregate food category level captured with the receipt data. However, further research using the method with a representative sample of households will yield findings about food expenditures, food sources and food types that can be generalized more broadly than is possible with the present data.

The comparison of week to total correlations for food categories suggests that a two-week receipt data collection period might provide adequate reliability to characterize the household food purchases. However, further research is needed to examine more closely the reliability within specific food categories, and to determine the optimal length of receipt data collection to provide reliable estimates of food category purchases within households. Seasonality also needs to be examined in consideration of the length of time and time of year receipt collection is implemented. Seasonality may not only affect food categories purchased (e.g. fruits and vegetables) but also affect food sources selected (e.g. higher frequency of eating-out during the summer or winter months, during vacations or holidays). However, in the present data, seasonality was not associated with the amounts or types of foods purchased that were captured by the receipt data.

The present study had several limitations that need to be addressed in follow-up research using the receipt methodology. First, the sample was motivated and complied well with the receipt collection and annotation protocol. Participant burden was moderately high, and participants received a financial incentive for compliance. The method may be less feasible with less motivated samples or without the financial incentive. However, when considering the use of the receipt methodology with population-based samples, it should be noted that other national consumer expenditure surveys conducted with general population have achieved acceptable compliance using modest financial incentives [27-30]. Second, a more representative sample will provide more generalizable information about the proportion of spending from home and eating out sources and for specific food categories. The present study specifically recruited households with at least one adult and one child, and households self-selected for interest in a weight gain prevention research study. Compared to national census statistics, the sample was comprised of more married (65% versus 51% nationally), well educated (26% greater than college degree versus 9% nationally) and higher income people (65% income > = US$50,000 versus 50% nationally)[31]. Third, expanding the store food and beverage categories will provide a broader perspective on the household food purchasing patterns. A wider range of food categories would not be too difficult to collect, annotate or code with slight modifications in the data collection protocol. This would afford a more complete examination of household food purchasing patterns and their associations with important variables such as income, family configuration and education. Finally, the receipt methodology, similar to other measures of dietary intake at the individual level, relies on self-report. The completeness of the receipt reporting, and the estimates of quantities (i.e. portion sizes at restaurants) is subject to error in reporting and social desirability influences. It is well-documented that individuals are not accurate at estimating portion sizes, and that individual characteristics are associated with under-reporting of dietary intake [32,33]. The receipt collection methodology avoids some of these errors by providing an objective measure of package sizes and quantities for most items purchased at stores and at chain restaurants with standard portion sizes. However, portion sizes from restaurants without standard portion size information available are subject to participant errors in reporting. Receipts are thought to temper the social desirability around reporting intake of certain foods viewed as less healthful because receipts are typically comprised of a list of foods and no identification of who was consuming each food item. However, under-reporting of receipts may still occur as the result of fatigue over a several week data collection period, or from the primary household shopper or other household members forgetting to collect receipts or report a food purchase or intentionally omitting certain food or beverage purchases. While it is possible to validate food purchases from direct observation, this approach may not be feasible because one or more observers would need to directly observe every household member for a period of several days. Reactivity is also a concern in that household members might alter their shopping habits during the observation period. The most feasible method of validation might be comparison with other household food purchase and availability measures, such as home food inventories and bar code scanners. However, these methods are less well designed to capture eating out food purchases. Ecological momentary assessment (EMA) measures could also be collected to measure each individual's food purchases during a defined time period [34]. However, the problem of omission, forgetting and fatigue are still an issue with EMA methods.

## Conclusion

In conclusion, annotated receipts can provide a measure of HFPB that is useful to examine in relation to household and individual demographic, dietary and behavioral characteristics. The household level food purchase behavior captured by the annotated receipts provides an important link both upward to the neighborhood food environment and downward to the individual dietary behavior. When combined with data from other levels of measurement, measures of HFPB can help improve understanding of how neighborhood food sources affect household food purchases, and how household food purchases affect individual dietary intake and food choices.

## Competing interests

The authors declare that they have no competing interests.

## Authors' contributions

SAF designed the study, developed the data collection protocol, supervised the data collection, participated in the data analysis, was the lead author of the manuscript. MW directed the data analysis and co-authored the manuscript, NM created the database, conducted data analysis, and co-authored the manuscript. STS and EW implemented the data collection protocol, coded the data and co-authored the manuscript. All authors read and approved the final manuscript.
